# Genomic Characteristics of an Extensive-Drug-Resistant Clinical *Escherichia coli* O99 H30 ST38 Recovered from Wound

**DOI:** 10.5812/ijpr-143910

**Published:** 2024-03-09

**Authors:** Ali A Dashti, Leila Vali, Sara Shamsah, Mehrez Jadaon, Sherief ElShazly

**Affiliations:** 1 Department of Medical Laboratory Sciences, Health Sciences Center, Faculty of Allied Health Sciences, Kuwait University, Kuwait; 2 School of Education and Applied Science, University of Gloucestershire, Cheltenham, UK; 3 Brescia University, Owensboro, KY, USA

**Keywords:** Whole Genome Sequencing, Colistin Resistance, Virulence Factors, Antimicrobial Resistance, Insertion Sequences

## Abstract

**Background:**

Antibiotic-resistant *Escherichia coli* is one of the major opportunistic pathogens that cause hospital-acquired infections worldwide. These infections include catheter-associated urinary tract infections (UTIs), ventilator-associated pneumonia, surgical wound infections, and bacteraemia.

**Objectives:**

To understand the mechanisms of resistance and prevent its spread, we studied *E. coli* C91 (ST38), a clinical outbreak strain that was extensively drug-resistant. The strain was isolated from an intensive care unit (ICU) in one of Kuwait's largest hospitals from a patient with UTI.

**Methods:**

This study used whole-genome sequencing (Illumina, MiSeq) to identify the strain's multi-locus sequence type, resistance genes (ResFinder), and virulence factors. This study also measured the minimum inhibitory concentrations (MIC) of a panel of antibiotics against this isolate.

**Results:**

The analysis showed that *E. coli* C-91 was identified as O99 H30 ST38 and was resistant to all antibiotics tested, including colistin (MIC > 32 mg/L). It also showed intermediate resistance to imipenem and meropenem (MIC = 8 mg/L). Genome analysis revealed various acquired resistance genes, including *mcr*-1, *bla*_CTX-M-14_, *bla*_CTX-M-15_, and *bla*_OXA1_. However, we did not detect *bla*_NDM_ or *bla*_VIM_. There were also several point mutations resulting in amino acid changes in chromosomal genes: *gyr*A, *par*C, *pmr*B, and *amp*C promoter. Additionally, we detected several multidrug efflux pumps, including the multidrug efflux pump *mdf*(A). Eleven prophage regions were identified, and PHAGE_Entero_SfI_NC was detected to contain ISEc46 and ethidium multidrug resistance protein E (*emr*E), a small multidrug resistance (SMR) protein family. Finally, there was an abundance of virulence factors in this isolate, including fimbriae, biofilm, and capsule formation genes.

**Conclusions:**

This isolate has a diverse portfolio of antimicrobial resistance and virulence genes and belongs to ST38 O99 H30, posing a serious challenge to treating infected patients in clinical settings.

## 1. Background

Multi-drug resistant *Escherichia coli* are opportunistic pathogens causing hospital-acquired infections worldwide. These infections include catheter-associated urinary tract infections, ventilator-associated pneumonia, surgical wound infections, and bacteraemia. They often carry resistance genes to antibiotics, such as β-lactams and fluroquinolone, that are commonly used for treatment. Genes encoding extended-spectrum β-lactamases (ESBLs) are often found on mobile genetic elements (MGEs) and are harbored within transposons or insertion sequences, thereby facilitating their spread to other strains. The most prevalent and dominant ESBL gene found in *Enterobacteriaceae* isolated from humans and food-producing animals is *bla*_CTX-M-15 _(1). Recently, a major concern has been the resistance to colistin, a polymixin, one of the last antibiotics in use after others failed. Colistin resistance gene *mcr*, which currently has ten variants, is usually found on plasmids of various incompatibility groups (IncX4, IncI2, and IncHI2) and often coexists with ESBLs (2, 3). In addition to ESBL genes, macrolide, tetracycline, aminoglycoside, fluoroquinolone, and carbapenem resistance genes can also coexist in a colistin-resistant isolate, limiting treatment options for hospitalized patients. 

Plasmid (*mcr*)- and chromosomal-mediated colistin resistance involve mutations in genes encoding enzymes that are associated with outer membrane modification of LPS by encoding a phosphoethanolamine transferase that catalyzes the addition of a phosphoethanolamine moiety to lipid A (3, 4), such as the *pmr*C and *pmr*E and the *pmr*HFIJKLM operon (4). Previous studies on *E. coli* have revealed that mutations in the sensor histidine kinase *pmr*B are an important mechanism of colistin resistance, leading to the constitutive production of the enzymes ArnT and EptA that add a positive charge (4-amino-4-deoxy-L-arabinose and phosphoethanolamine, respectively) to the phosphate groups of lipid A and reducing the affinity of colistin to bind to lipid A (3, 4).

To plan effective treatment guidelines, it is crucial to understand the mechanisms of resistance and epidemiology of multidrug-resistant (MDR) *E. coli* in both the community and hospitals. Given the burden of diseases caused by *E. coli* and its significant public health concern, hospitals should continuously monitor their antimicrobial treatment efficacy. Whole-genome sequencing (WGS)-based in silico approaches are valuable tools in gene analysis of outbreak strains that offer detailed epidemiological investigation and tracing of pathogens (5). In this study, we used WGS to characterize *E. coli* C91 (ST38), an extensively drug-resistant clinical outbreak strain isolated from patient zero in the intensive care unit (ICU) of one of the largest hospitals in Kuwait, with the intention of successfully treating the patients and containing its spread.

## 2. Methods

### 2.1. Sample Collection

A clinical *E. coli* isolate C91 was isolated from a post-surgical wound of a 53-year-old male admitted to ward 8/ICU (26/11/2016) and was initially identified by VITEK 2 ID system (bioMérieux, Marcy-l’Etoile, France). This patient was named patient zero. 

### 2.2. Antibiotic Sensitivity Testing

Antimicrobial sensitivity testing was carried out according to the Clinical and Laboratory Standards Institute (2020) (6). The minimum inhibitory concentrations (MICs) were determined for aminoglycosides, chloramphenicol, tetracycline, β-lactams, including carbapenems, and in combination with β-lactam inhibitors, ciprofloxacin, erythromycin, trimethoprim, gentamycin, and colistin. The MIC (μg/mL) against a panel of antibiotics were determined using E test (bioMérieux, Marcy-l’Etoile, France). For colistin, the agar dilution method was used (6). 

### 2.3. Whole-Genome Sequencing Analyses

Genomic deoxyribonucleic acid (DNA) was extracted using QIAamp^®^ DNA Mini Kit (Qiagen, Hilden, Germany) and quantified by the NanoDrop-800 spectrophotometer (Thermo Fisher Scientific, Wilmington, NC, USA) according to the manufacturer’s instructions. The WGS was performed by MicrobesNG, University of Birmingham, UK (https://microbesng.uk) using the Illumina MiSeq^®^ sequencer platform. The reads were trimmed using Trimmomatic, and the quality was assessed by MicrobesNG’s in-house scripts combined with the following software packages: SAMtools (Sequence, Alignment/Map), Bedtools, and bwa-mem (Burrows-Wheeler Aligner). All statistics are based on contigs of size ≥ 500 bp unless otherwise noted. The trimmed data were assembled using the SPAdes algorithm assembler (version: 3.7.1); this de novo assembly of the quality-controlled reads was assembled to create a draft genome sequence, and variant calling was performed using VarScan. An automated annotation was performed using Prokka (version 1.13.3). The WGS of the isolate was submitted to Genbank Accession: SAMN10105215, ID: 10105215 (sample name: *Escherichia coli* strain Kuwait C-91).

### 2.4. In Silico Molecular Analysis

For in silico WGS analysis, the assembled sequences were uploaded onto the Center for Genomic Epidemiology to identify the following: ResFinder v4.3.3, ResFinderFG 2.0, KmerResistance 2.2 (7), PathogenFinder1.1 (8), VirulenceFinder 2.0 (9-11), multilocus sequence typing 2.0 (12), PlasmidFinder 2.1 (13), MGE v1.0.3 (14), SerotypeFinder 2.0 (15), FimTyper 1.0 (16), and 2.1 (13), CHTyper 1.0 (17), CARD 2020 annotation (18), Pfam (InterPro 95.0), VirSorter2 version 2.2.4 (https://u.osu.edu/viruslab/). The presence of insertion sequences was confirmed using ISFinder (19). Proksee CGView.js server was used for genome assembly, annotation, and visualization and provided a complete genome CGView/Proksee map JSON file (20).

### 2.5. Detection of Phages from WGS 

Phaster tool (21) was used to identify prophage sequences. This tool classifies the phages into three classes (intact, questionable, and incomplete) based on their completeness (phage score). Additionally, by using the Proksee server, phages were identified with the VirSorter2 2.2.4 tool and were screened for antimicrobial resistance genes using the basic local alignment search tool (BLAST). 

## 3. Results

### 3.1. Description of the Isolate and Mapping Summary

**Figure 1.**
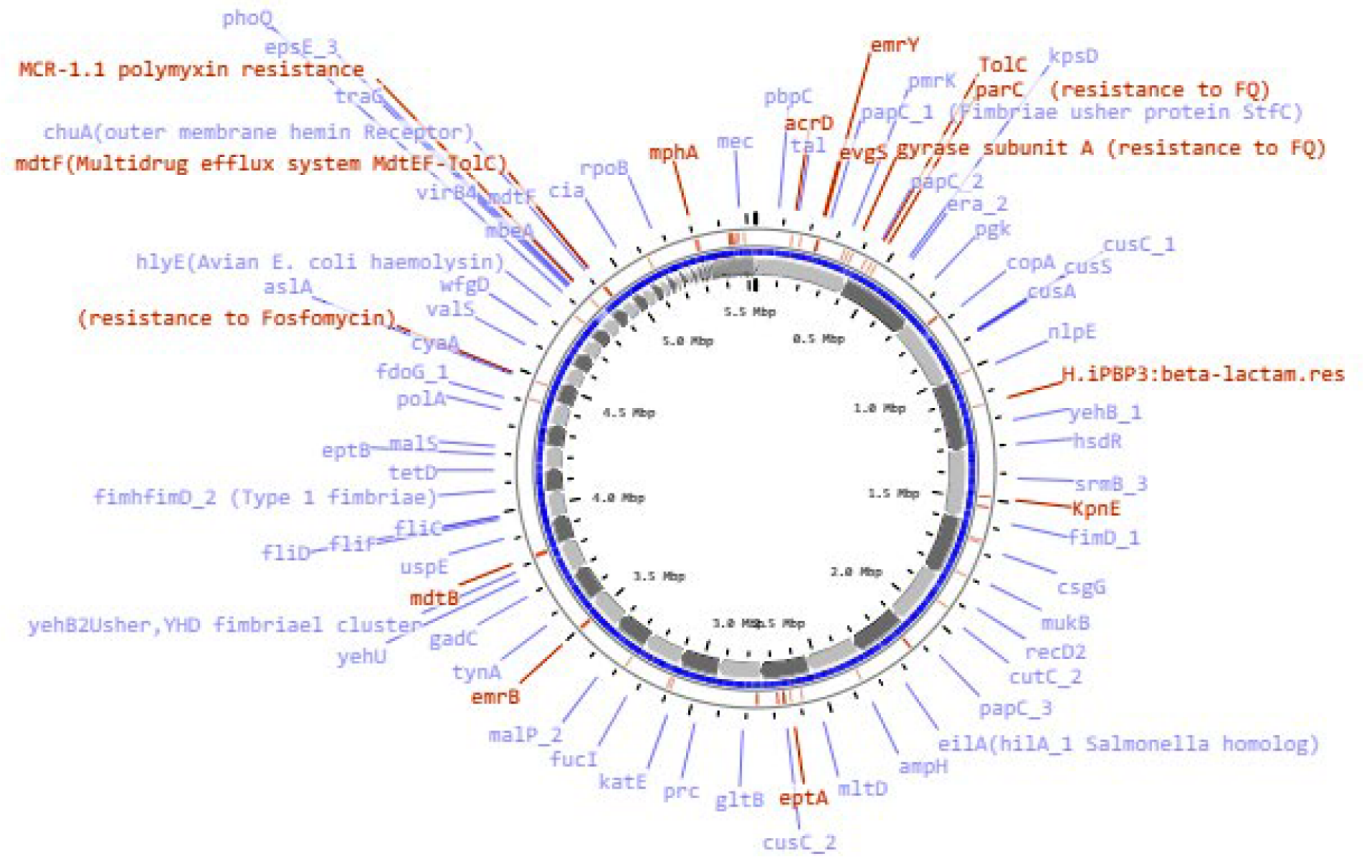
The gene map of *E. coli* C91 with labels showing the resistance (red) and virulence (blue) genes.

**Figure 2.**
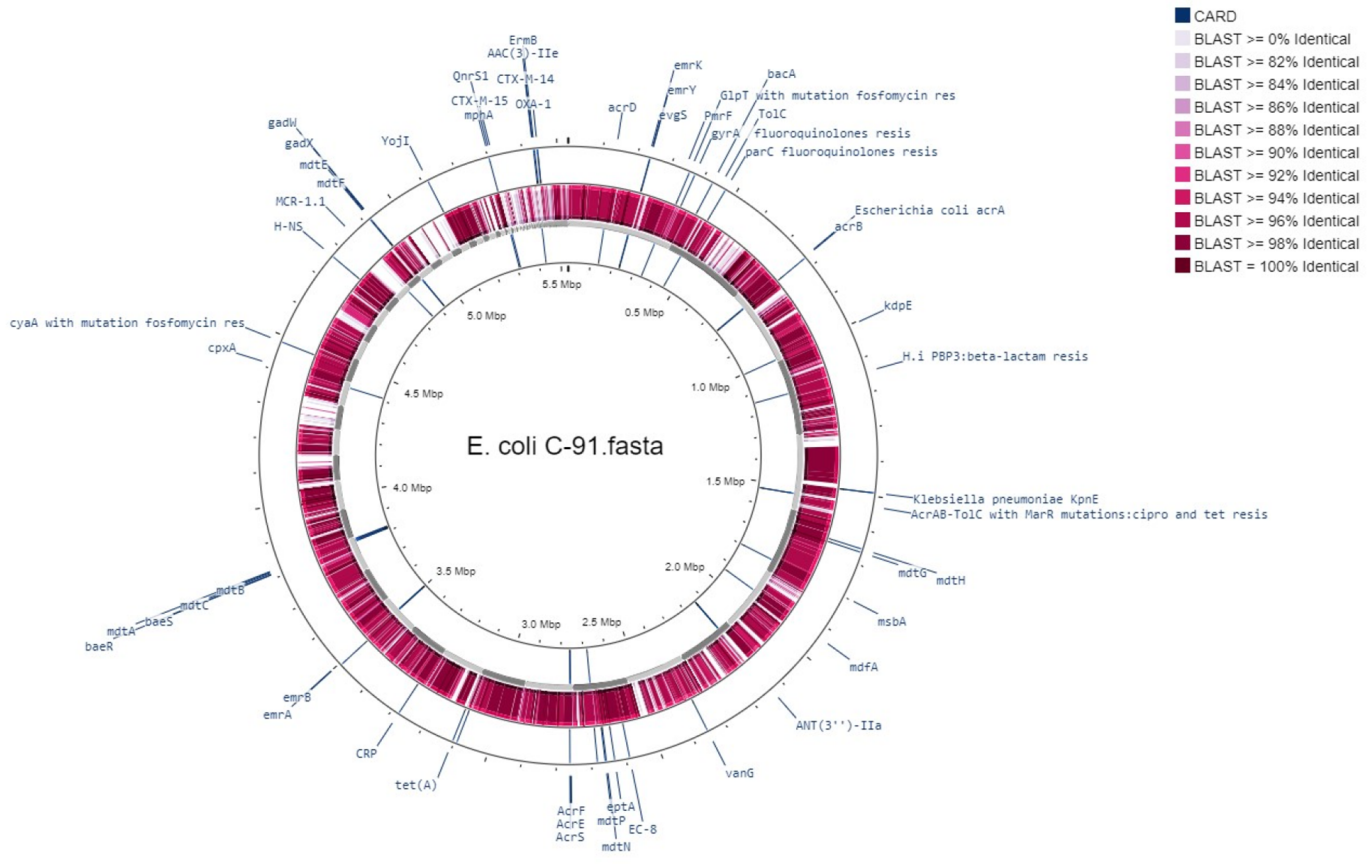
Color existing basic local alignment search tool (BLAST) features by percent identity and sort BLAST tracks by similarity, *E. coli* C91 backbone vs. *E. coli* K12 MG1655 (GenBank: U00096.2).

The bacterial strain C91 was identified as *E. coli* O99 H30 ST38 according to two different schemes, Warwick and Pasteur Institute (Appendix 1). The draft genome was annotated using RAST (Table 1) and revealed a linear chromosome consisting of 5 532 235 base pairs, with 4 964 coding sequences, 87 transfer RNA (tRNA) genes, and several proteins with functional assignments. The genome was assembled using the SPAdes assembler (version: 3.7.1) from trimmed data, producing an N50 quality value of 181 117 and a L50 of 11, with an N75 of 97 722 and L75 of 20. The sample had a mapping rate of 76.81% against the reference genome (without Ns), with an average depth of 76.96X and over 90.93% coverage of more than 1X, a result that falls within the normal range. The genome mapping of antimicrobial resistance and virulence factors is shown in Figure 1, and the comparison of *E. coli* C91 to *E. coli* K12 MG1655 (GenBank: U00096.2) using NCBI and Proksee software is presented in Figure 2. 

**Table 1.** Summary of the Statistics of the Assembled Genome of *E. coli* C91

*E. coli* C91	Values
**Genome size (bp)**	5 532 235
**Total length of the genes (bp) **	4 529 853
**GC content %**	51.45
**Number of genes**	4 964
**% of genome (genes)**	86.67
**Gene average length (bp)**	913
**Gene internal length**	696 465
**Gene internal GC content**	43.87
**% of genome (internal)**	13.33
**Average depth **	76.96X
**Contigs**	183
**Largest contig**	399 443
**Genome coverage**	90.93%
**GC%**	50.42
**N50**	181 117
**N75**	97 722
**N90**	66 020
**L50**	11
**L75**	20
**sRNAs**	78
**tRNAs**	87

### 3.2. Plasmids and MGEs

Five plasmids IncY, IncI2(Delta), IncFIC(FII), IncI1-I(Alpha), and IncFIBIncF and 17 MGEs, including Tn7, were detected harboring antibiotic resistance genes. Their locations are shown in Table 2. 

**Table 2.** Plasmids Identified in *E. coli* C91

Plasmid	Contig	Position in Contig	Coverage %	Identity %	Accession No.	Resistance Genes/Phage
**IncY**	NODE_22_length_94041_cov_7.78437	24344..25108	100	99.74	K02380	Circular phage/ PHAGE_Salmon_SJ46_NC_031129(89)
**IncI2(Delta)**	NODE_29_length_60972_cov_14.1908	4841..5156	100	98.42	AP002527	mcr-1.1
**IncFIC(FII)**	NODE_32_length_48168_cov_7.4304	2747..3243	99.4	94	AP001918	-
**IncI1-I(Alpha)**	NODE_33_length_45415_cov_9.87703	15412..15553	100	99.3	AP005147	-
**IncFIB**	NODE_53_length_5159_cov_9.67925	2548..3229	100	97.65	AP001918; CP053724	-

### 3.3. Antibiotic Sensitivity Testing and Resistance Genes

*E. coli* C91 was resistant to all antibiotics tested, including aminoglycosides, chloramphenicol, tetracycline, β-lactams (both alone and in combination with β-lactam inhibitors), ciprofloxacin, erythromycin, trimethoprim, gentamycin, and colistin. The MIC for these antibiotics was greater than 32 mg/L. It also demonstrated intermediate resistance to imipenem and meropenem with an MIC of 4 mg/L. The analysis of the genome of *E. coli* C91 revealed the presence of 200 antibiotic-resistance genes, including efflux pump complexes and antibiotic target protection proteins, as confirmed by CARD annotations. The genome analysis also revealed the presence of *mcr*-1, *bla*_CTX-M-14_, *bla*_CTX-M-15_, and *bla*_OXA-1 _genes, but not *bla*_NDM_ or *bla*_VIM_. The bacterium was observed to have acquired resistance genes, including *aac*(3)-IIa, *aac*(6')Ib-cr, *aad*A1, *qnr*S1, *cat*B4, *tet*A, *mph*A, *erm*B, and *dfr*A1, as shown in Table 3 (and Appendix 2) and Figure 1. Point mutations were also detected in chromosomal resistance genes, including *gyr*A, *par*C, and *pmr*B, leading to changes in amino acids, as shown in Table 4. The results of some of these mutational modifications are not clear.

**Table 3.** Antimicrobial Resistance Genes and Their Phenotypic Characteristics Identified in *E. coli* C91

Gene	Phenotype	Position in Contig/MGE, Plasmid	Coverage %	Identity	Accession
***D-alanine--** **D-alanine** ** ligase ** **van_ligase***	Cycloserine	NODE_4_length_294518_cov_34.7303_119741_118821	100	99.02	KF628564.1
***D-** **alanyl** **-D-alanine carboxypeptidase, none enzyme β_lactam** **resistance***	Penicillin	NODE_7_length_249411_cov_31.6868_90740_91963	99	99.75	BDB50754.1
***ant(3'')-** **Ia** **, (aadA1)***	Spectinomycin, streptomycin	NODE_8_length_222074_cov_39.2814_40651_39863/Tn7	100	100	JQ480156
***dfrA1***	Trimethoprim	NODE_8_length_222074_cov_39.2814_41801_41328/Tn7	100	100	X00926
***Multidrug resistance protein ** **MdtL***		NODE_8_length_222074_cov_39.2814_62182_61007	100	100	WP_000086009.1
***Multidrug efflux MFS transporter ** **EmrD***		NODE_8_length_222074_cov_39.2814_102857_101673	100	99	WP_097336506.1
***van_ligase***	D-cycloserine	NODE_9_length_221455_cov_34.9789_34311_35405	100	99.02	KF628791.1
***β-lactamase***	Piperacillin	NODE_10_length_210474_cov_36.1065_48903_50069	100	99.83	KU607300.1
***emrE** ** (SMR protein family)***	Ethidium multidrug resistance	NODE_12_length_170122_cov_27.8913/ISEc46			
***tet** **(A)***	Tetracycline, oxytetracycline, doxycycline; minocycline	NODE_13_length_161358_cov_37.5202_2716_3915/Tn5403	100; 100	100; 99.85	AJ517790; JX009293.1; GQ343144.1
***sitABCD***	Hydrogen peroxide	NODE_16_length_138200_cov_28.3328_4692_1243	99.59	97.48	AY598030
***Multidrug efflux system ** **MdtABC-TolC***		NODE_17_length_131052_cov_29.4018_123533_115966	100	100	CP128875.1
***mcr-1.1***	Polymyxin, colistin	NODE_29_length_60972_cov_14.1908_47111_45486/ Incl2(Delta)	100	100	KP347127; OM179755.1
***qnrS1***	Ciprofloxacin	NODE_43_length_11726_cov_19.4543_6035_5379/ISKpn19	100	100	AB187515
***mph(A) (Macrolide phosphotransferase)***	Azithromycin, telithromycin, erythromycin, spiramycin	NODE_43_length_11726_cov_19.4543_197_1102/ISKpn19	100	100	D16251
***blaCTX-M-15 (Class A)***	Ticarcillin, aztreonam, ampicillin, amoxicillin, piperacillin, ceftazidime, cefotaxime, ceftriaxone, cefepime	NODE_43_length_11726_cov_19.4543_11551_10676/ISKpn19	100	100	AY044436, GQ343005.1
***bla***_**CTX-M-14**_ **; ** ***(Class A, bla***_**CTX-M-14a-like**_ **)**	Ticarcillin, aztreonam, ampicillin, amoxicillin, piperacillin, ceftazidime, cefotaxime, ceftriaxone, cefepime	NODE_65_length_3010_cov_7.26882_2841_1966/IS102	100; 100	100; 99.89	AF252622; KU544013.1
***aminoglycoside N(3')-acetyltransferase III gene*** **; ** ***aac** **(3)-** **IIe***	Gentamicin	NODE_66_length_2854_cov_39.711_171_1031/ISKpn19	100	100	GQ343134.1; CP125071; HCQ1792082.1
***aac** **(3)-** **IIa***	Gentamycin, tobramycin	NODE_66_length_2854_cov_39.711_171_1031//ISKpn19	100	100	CP023555
***erm** **(B)***	Macrolide, lincosamide, streptogramin, quinupristin/dalfopristin	NODE_67_length_2837_cov_7.75646_420_1157	100; 100	99.73; 99.86	JN899585; CP082057
***aac** **(6')-** **Ib-cr***	Fluoroquinolone, ciprofloxacin, dibekacin, sisomicin, netilmicin, amikacin, tobramycin	NODE_70_length_2440_cov_46.4838_174_773	100	100	DQ303918; GQ342986.1
***bla***_**OXA-1**_	Carbenicillin, ampicillin, amoxicillin, piperacillin, cefepime, ampicillin+clavulanic acid, amoxicillin+clavulanic acid, piperacillin+tazobactam	NODE_70_length_2440_cov_46.4838_859_1734	100	100	HQ170510; MN340011.1
***catB3***	Chloramphenicol	NODE_70_length_2440_cov_46.4838_1872_2420	70	100	U13889; AJ009818; KU544029.1

**Table 4.** Chromosomal Point Mutations and Their Phenotypic Characteristics Identified in *E. coli* C-91

Mutation	Nucleotide Change	Amino Acid Change	PMID	Notes
***gyrA** ** p.S83L***	TCG → TTG	S → L	8891148, 2168148, 12654733, 12654733	
***gyrA** ** p.D87N***	GAC → AAC	D → N	12654733, 12654733, 12654733, 22878251, 12654733, 1850972	D87G or D87Y confer resistance to nalidixic acid only, if occurring alone. Unknown phenotype if D87H occurs alone
***gyrA:p.D678E***	GAC → GAA	D → E	Phenotype not found in database	Unknown phenotype
***parE** ** p.S458A***	TCG → GCG	S → A	14506034, 28598203	Unknown phenotype if S458T or S458A occurs alone. Nalidixic acid and ciprofloxacin resistance when associated with *gyr*A mutations
***parC** ** p.S57T***	AGC → ACC	S → T	14510643	Unknown phenotype if S57T occurs alone. Nalidixic acid and ciprofloxacin resistance when associated with *gyr*A
***parC** ** p.S80I***	AGC → ATC	S → I	8851598, 8851598, 21856834-20638608, 8524852, 25631675, 25631675, 25631675	Unknown phenotype if each mutation occurs alone. Nalidixic acid and ciprofloxacin resistance when associated with *gyr*A mutations
***parC:p.E62K***	GAA → AAG	E → K	Phenotype not found in database	Unknown phenotype
***parC:p.D475E***	GAT → GAA	D → E	Phenotype not found in database	Unknown phenotype
***parC:p.K200N***	AAA → AAT	K → N	Phenotype not found in database	Unknown phenotype
***parC:p.L344R***	CTG → CGG	L → R	Phenotype not found in database	Unknown phenotype
***parC:p.D197E***	GAC → GAG	D → E	Phenotype not found in database	Unknown phenotype
***parC:p.D309E***	GAT → GAG	D → E	Phenotype not found in database	Unknown phenotype
***ampC** ** promoter:p.R24***	CGA → TGA	R → *	Phenotype not found in database	Unknown phenotype
***pmrB:p.H2R***	CAT → CGT	H → R	Phenotype not found in database	Unknown phenotype
***pmrB:p.D283G***	GAC → GGC	D → G	Phenotype not found in database	Unknown phenotype
***pmrB:p.Y315F***	TAT → TTT	Y → F	Phenotype not found in database	Unknown phenotype

### 3.4. Virulence Factors

This isolate has an abundance of virulence factors shown in Table 5 and Figure 1, including fimbriae, biofilm, and capsule formation genes.

**Table 5.** Virulence Factors, Protein Function, and Their Position in Contig

Virulence Factor	Identity	Query/Template Length	Contig	Position in Contig	Protein Function	Accession Number
***AslA***	98.31	1656/1656	NODE_24_length_92227_cov_34.925	37443..39098	Contributing to the invasion of brain microvascular endothelial cells	CP022686
***aamR:FN554766***	99.84	645/645	NODE_2_length_314467_cov_36.7095	209279..209923	Not known	
***Air***	95.16	4604/4605	NODE_8_length_222074_cov_39.2814	120721..125324	Enteroaggregative immunoglobulin repeat protein	CP003034
***Anr***	96.24	213/213	NODE_32_length_48168_cov_7.4304	4169..4381	AraC negative regulator	AL391753
***capU***	99.91	1089/1089	NODE_38_length_25756_cov_32.6998	7151..8239	Hexosyltransferase homolog	CU928145
***chuA***	100	1983/1983	NODE_30_length_56813_cov_40.9025	37851..39833	Outer membrane hemin receptor	UFZU01000002
***Cia***	100	147/147	NODE_33_length_45415_cov_9.87703	8729..8875	Colicin	QMGM01000002
***csgA***	92.98	456/456	NODE_6_length_255487_cov_30.7367	82846..83301	curlin major subunit CsgA (biofilm)	CP069646
***eilA***	98.65	1698/1698	NODE_8_length_222074_cov_39.2814	131902..133599	Salmonella HilA homolog	FN554766
***espY2:000868321***	94.56	570/570	NODE_4_length_294518_cov_34.7303	145342..145911	Not known	
***fdeC***	92.15	4214/4254	NODE_9_length_221455_cov_34.9789	120658..124871	intimin-like adhesin FdeC	AP010953
***fimH***	100	489/489	NODE_20_length_97722_cov_41.3206	15817..16305	Type 1 fimbriae	NA
***Gad***	99.1	1116/1401	NODE_96_length_1120_cov_51.0514	1..1116	Glutamate decarboxylase	FN554766
***hlyE***	98.91	918/918	NODE_27_length_69431_cov_30.2889	62576..63493	Avian *E. coli* haemolysin	ECU57430
***Hra***	95.01	741/741	NODE_20_length_97722_cov_41.3206	95294..96034	Heat-resistant agglutinin	CP040456
***Hra***	100	792/792	NODE_2_length_314467_cov_36.7095	219779..220570	Heat-resistant agglutinin	CP043942
***Iss***	100	294/294	NODE_40_length_20447_cov_25.9281	19924..20217	Increased serum survival	CP001846
***kpsE***	100	1149/1149	NODE_2_length_314467_cov_36.7095	155149..156297	Capsule polysaccharide export inner-membrane protein	AAMK02000004
***kpsMII_K5***	100	777/777	NODE_2_length_314467_cov_36.7095	141362..142138	Polysialic acid transport protein; Group 2 capsule	MG739441
***neuC***	100	1176/1176	NODE_2_length_314467_cov_36.7095	145757..146932	Polysialic acid capsule biosynthesis protein	JJLW01000144
***nlpI***	99.77	885/885	NODE_11_length_181117_cov_35.2668	107595..108479	lipoprotein NlpI precursor	CP000243
***sitA***	100	915/915	NODE_16_length_138200_cov_28.3328	3778..4692	Iron transport protein	HG977190
***terC***	98.46	714/714	NODE_13_length_161358_cov_37.5202	84643..85356	Tellurium ion resistance protein	CP000468
***terC***	98.54	959/966	NODE_11_length_181117_cov_35.2668	173664..174622	Tellurium ion resistance protein	MG591698
***traJ***	98.55	690/690	NODE_32_length_48168_cov_7.4304	34241..34930	Protein TraJ (positive regulator of conjugal transfer operon)	AF550679
***traT***	100	777/777	NODE_32_length_48168_cov_7.4304	13597..14373	Outer membrane protein complement resistance	AAJW02000025
***yehA***	95.85	1035/1035	NODE_17_length_131052_cov_29.4018	90990..92024	Outer membrane lipoprotein, YHD fimbriae cluster	CP042934
***yehB***	97.5	2481/2481	NODE_17_length_131052_cov_29.4018	88494..90974	Usher, YHD fimbriae cluster	CP042934
***yehC***	96.3	675/675	NODE_17_length_131052_cov_29.4018	87804..88478	Chaperone, YHD fimbriae cluster	CP042934
***yehD***	97.24	543/543	NODE_17_length_131052_cov_29.4018	87181..87723	Major pilin subunit, YHD fimbriae cluster	CP042934

### 3.5. Phage Analysis

Eleven prophage regions were identified in *E. coli* C91, from contig 1 - 45, using the Phaster tool (Table 6 Appendix 3). Out of these regions, three are intact, seven are incomplete, and one is questionable. However, when the VirSorter2 2.2.4 tool was used in Proksee software, phages were also picked up from nodes 46-183 (Table 6). One of the intact phages is PHAGE_Salmon_SJ46_NC_031129(89)(IncY), located on NODE_22_length_94041_cov_7.78437. On NODE_12, PHAGE_Entero_SfI_NC_027339(6) (partial sequence) was detected, containing ISEc46 and *emr*E (ethidium multidrug resistance protein E), an SMR protein family.

**Table 6.** Phage Analysis with Phaster Tool Indicative of the Regions Containing Phages ^a^

	Region	Region Length (kb)	Completeness	# Total Proteins	Most Common Phage	GC %
**NODE_5_length_285039_cov_26.0736**	1	44.1	Intact	54	PHAGE_Entero_P88_NC_026014(33)	52.82
	2	16.3	Questionable	24	PHAGE_Salmon_118970_sal3_NC_031940(4)	50.68
**NODE_12_length_170122_cov_27.8913**	3	26.8	Incomplete	24	PHAGE_Entero_SfI_NC_027339(6)	45.39
**NODE_18_length_114548_cov_26.6919**	4	26.9	Incomplete	21	PHAGE_Shigel_POCJ13_NC_025434(6)	45.95
**NODE_19_length_110482_cov_29.551**	5	27.8	Incomplete	31	PHAGE_Entero_phiP27_NC_003356(13)	48.50
**NODE_22_length_94041_cov_7.78437**	6	92.5	Intact	117	PHAGE_Salmon_SJ46_NC_031129(89)	48.07
**NODE_34_length_38724_cov_8.49753**	7	9.1	Incomplete	14	PHAGE_Rhodoc_RGL3_NC_016650(1)	56.85
**NODE_39_length_24718_cov_29.6434**	8	24.3	Intact	28	PHAGE_Pseudo_phiPSA1_NC_024365(7)	48.89
**NODE_40_length_20447_cov_25.9281**	9	19.9	Incomplete	20	PHAGE_Entero_lambda_NC_001416(19)	56.34
**NODE_43_length_11726_cov_19.4543**	10	8.9	Incomplete	11	PHAGE_Microc_MaMV_DC_NC_029002(2)	51.76
**NODE_45_length_8140_cov_11.3055**	11	7.6	Incomplete	9	PHAGE_Escher_RCS47_NC_042128(3)	48.13

^a^ Region: The number assigned to the region. Region length: The length of the sequence of that region (in bp). Completeness: A prediction of whether the region contains an intact or incomplete prophage. # Total proteins: The number of ORFs present in the region. Most common phage: The phage(s) with the highest number of proteins most similar to those in the region. GC %: The percentage of GC nucleotides of the region.

## 4. Discussion

Colistin-resistant *E. coli* is one of the most important nosocomial pathogens with limited treatment options. The present study characterized a multi-drug resistant clinical *E. coli* (C-91) isolate causing complications in the ICU of one of the largest hospitals in Kuwait. This isolate has a diverse collection of genes conferring resistance to an array of antimicrobial agents. It contains *bla*_CTX-M-15_, the most dominant ESBL (22), and *bla*_CTX-M-14_ (23), in addition to other important resistance genes, including *aad*A1, *aac*(3)-IIa, *aac*(6')-*Ib-cr*, *bla*_OXA-1_, *mcr*-1.1, *mph*(A), *erm*(B), *cat*B3, *qnr*S1, *tet*(A), *dfr*A1, and *mph*A (the most common azithromycin resistance gene detected in *E. coli*). It encodes for resistance enzyme MPH(2')-I, which inactivates 14-membered macrolides (e.g., erythromycin, telithromycin, roxithromycin) over 16-membered macrolides (e.g.tylosin and spiramycin) (24). In this study, *aac*(3)-IIa, *qnr*S1, *mph*(A), and *bla*_CTX-M-15 _genes were associated with insertion sequence (IS) ISKpn19. *bla*_CTX-M-14 _was associated with IS102, *tet*(A), *ter*C with Tn5403, and *ant(3'')-Ia*, (*aad*A1), *dfr*A1 with Tn7. In total, we identified 14 insertion sequences and transposons (Appendix 4). Insertion sequence elements can play an integral role in the transfer of these resistance genes and virulence factors in their surrounding regions (25).

*Escherichia coli* C91 also contains several multidrug efflux pumps, including the multidrug efflux pump *mdf*(A), which confers resistance to antibiotics, such as chloramphenicol, erythromycin, and fluoroquinolones (26). The present study also detected mutations in chromosomally encoded *gyr*A, *gyr*B, *par*C, *pmr*B, *amp*C, and *cya* genes causing resistance to fluoroquinolones, polymyxins, and fosfomycin. We did not detect *bla*_NDM_, *bla*_VIM_, nor *bla*_OXA-48_ in this isolate, althrough the MIC for imipenem was just below the cutoff point (MIC = 4). However, others have reported the prevalence of carbapenem resistance among *Enterobacteriaceae* in hospitals in Kuwait (27).

Antimicrobial resistance plasmids present in *E. coli* C91 comprise epidemic resistance plasmids IncFIB and IncFIC(FII), which can acquire resistance determinants and disseminate readily among *Enterobacteriaceae* and broad-range IncY, IncI2 (pMCR-1) carrying the *mcr*-1 gene. IncI1-I plasmids have been shown to propagate the resistance genes between different species (28). Therefore, this isolate has the potential to tolerate and resist conventional antibiotic therapies. 

The identification of *E. coli* clones in the fields of taxonomy and epidemiology is predicated on a combination of O- and H- antigens. These antigens are characterized by variations in the sugars present in the O unit and the linkages between O units (29). There are currently 185 O antigens, and the O99 antigen consists of four d-rhamnose moieties in the backbone and two d-glucose moieties in the side chain. The O-antigen is synthesized and transported by an ABC transporter-dependent process and is considered an important virulence factor, offering selective advantages in specific niches. Pathogenic clones are often found to have a higher incidence of certain O antigens (29, 30).

H-antigens (flagellins) are encoded by *fli*C genes, with 53 different serotypes of H-antigen identified (31). The diversity of H-antigens arises from lateral gene transfer and recombination of foreign DNA, generating alleles and antigenic variation (32). *Fim*H genes encode a type I fimbria that enables adherence and infects the epithelial urinary tract tissue expressed in uropathogenic *E. coli* (UPEC). *Fli*C genes encode proteins that promote successful host colonization and are involved in interleukin-6 (IL-6) and interleukin-8 (IL-8) release. *Fum*C genes encode a protein that catalyzes fumarate oxidation to malate during the oxidative TCA cycle under aerobic conditions. *Fum*C is required for *E. coli* fitness in vivo, and a loss of *Fum*C results in delayed growth during iron limitation (33-35).

The H30 subclone has been reported to be responsible for the clonal dissemination of ST131 *E. coli* (36). Therefore, it is proposed that H30 provides ST38 clones with the advantage of propagation. Since *E. coli* sequence type ST38 has become prominently associated with hospital- and community-acquired infections worldwide (37-39), it is crucial to identify the subclones to increase the chances of successful treatments.

In conclusion, *E. coli* C91 (ST38) O99 H30 is a high-risk and globally disseminated extraintestinal pathogenic (ExPEC) strain that can cause invasive infections and resist multiple antibiotic treatments. This study used WGS and in silico analysis to identify the molecular characteristics of this isolate. The obtained results showed that it contains genes encoding ESBLs that confer resistance to cephalosporins and other β-lactam antibiotics. Additionally, *E. coli* C91 (ST38) is resistant to macrolides, tetracyclines, aminoglycosides, and fluoroquinolones, making it extensively drug-resistant (XDR). Furthermore, it carries *mcr*-1 gene, which severely limits the treatment options. This isolate also encodes several virulence factors facilitating biofilm formation and adherence to tissues. Infections caused by XDR *E. coli* C91 (ST38) O99 H30 in the ICU might be life-threatening and require urgent treatment.

ijpr-21-1-127043-s001.pdf

## Data Availability

All data are available in publicly accessible databases under the accession numbers reported.
