# Supplementary material for: Genomic Characteristics of an Extensive-Drug-Resistant Clinical Escherichia coli O99 H30 ST38 Recovered from Wound
Source: Iran J Pharm Res. 2024 Mar 9;23(1):e143910. doi: 10.5812/ijpr-143910 (PMC11246641; doi:10.5812/ijpr-143910)
Supplement: ijpr-21-1-127043-s001.pdf [file ijpr-23-1-143910-s001.pdf]

Appendix 1. Serotyping results group *E. coli* C91 as O99 H30

Gene	*Serotype*	*Identity*	*Template/HSP length*	*Contig*		*Position in contig*	*Accession no.*
wzt	O99	100	1302/1302	NODE_26_length_73147_cov_31.1708		36020..37321	FJ940773
wzm	O99	100	795 / 795	NODE_26_length_73147_cov_31.1708		37323..38117	FJ940773
fliC	H30	99.94	1713 / 1713	NODE_19_length_110482_cov_29.551		41687..43399	AY250011

Appendix 2. Identification of resistance genes and their contig position in the genome of *E. coli* C91

**Resistance gene**	**Identity%**	**Alignment length/gene length**	**Position in reference**	**Contig or depth**	**Position in contig**	**Phenotype**	**Accession no.**	**Notes**
***aad*A1**	100	789 / 789	1...789	NODE_8_length_222074_cov_39.2814	39863...40651	streptomycin, spectinomycin	JQ480156	Alternative name *ant*(3")-Ia
***aac*(3)-IIa**	100	861 / 861	1...861	NODE_66_length_2854_cov_39.711	171...1031	gentamicin, tobramycin	CP023555	
***aac*(6')-Ib-cr**	100	600 / 600	1...600	NODE_70_length_2440_cov_46.4838	174...773	tobramycin, amikacin, dibekacin, netilmicin, sisomicin, fluoroquinolone,	DQ303918	MIC of ciprofloxacin does not always increase above ECOFF

						ciprofloxacin		PMID 16369542
***bla*$_{CTX-M-15}$**	100	876 / 876	1...876	NODE_43_length_11726_cov_19.4543	10676...11551	amoxicillin, ampicillin, cefepime, cefotaxime, ceftazidime, piperacillin, aztreonam, ticarcillin, ceftriaxone	AY044436	Class A
***bla*$_{CTX-M-14}$**	100	876 / 876	1...876	NODE_65_length_3010_cov_7.26882	1966...2841	amoxicillin, ampicillin, cefepime, cefotaxime, ceftazidime, piperacillin, aztreonam, ticarcillin, ceftriaxone	AF252622	Class A; Alternative name *bla*$_{CTX-M-14a}$, *bla*$_{CTX-M-18}$
***bla*$_{OXA-1}$**	100	831 / 831	1...831	NODE_70_length_2440_cov_46.4838	904...1734	amoxicillin, amoxicillin+clavulanic acid, ampicillin, ampicillin+clavulanic acid, cefepime, piperacillin, piperacillin+tazobactam	HQ170510	Class D; *OXA*-1-like; Alternative name *bla*$_{OXA-30}$
***mcr*-1.1**	100	1626 / 1626	1...1626	NODE_29_length_60972_cov_14.1908	45486...47111	colistin	KP347127	
***mph*(A)**	100	906 /	1...906	NODE_43_length_11726_cov	197...1102	erythromycin,	D16251	Macrolide

		906		_19.4543		azithromycin,		phosphotransferase
***erm*(B)**	99.73	738 / 738	1...738	NODE_67_length_2837_cov_7.75646	420...1157	lincomycin, clindamycin, erythromycin, quinupristin, pristinamycin, virginiamycin	JN899585	23S rRNA methylase; Azithromycin, Spiramycin resistance in Staphylococcus sp. and Streptococcus sp.; If inducible phenotype, patterns of spiramycin, lincomycin and clindamycin resistance may vary
***cat*B3**	100	442 / 633	1...442	NODE_70_length_2440_cov_46.4838	1872...2313	chloramphenicol	AJ009818 U13880	Chloramphenicol acetyltransferase
***qnr*S1**	100	657 / 657	1...657	NODE_43_length_11726_cov_19.4543	5379...6035	ciprofloxacin	AB187515	
***tet*(A)**	100	1200 / 1200	1...1200	NODE_13_length_161358_cov_37.5202	2716...3915	tetracycline, doxycycline	AJ517790	
***dfr*A1**	100	474 / 474	1...474	NODE_8_length_222074_cov_39.2814	41328...41801	trimethoprim	X00926	

Appendix 3: Phage analysis using Phaster tool

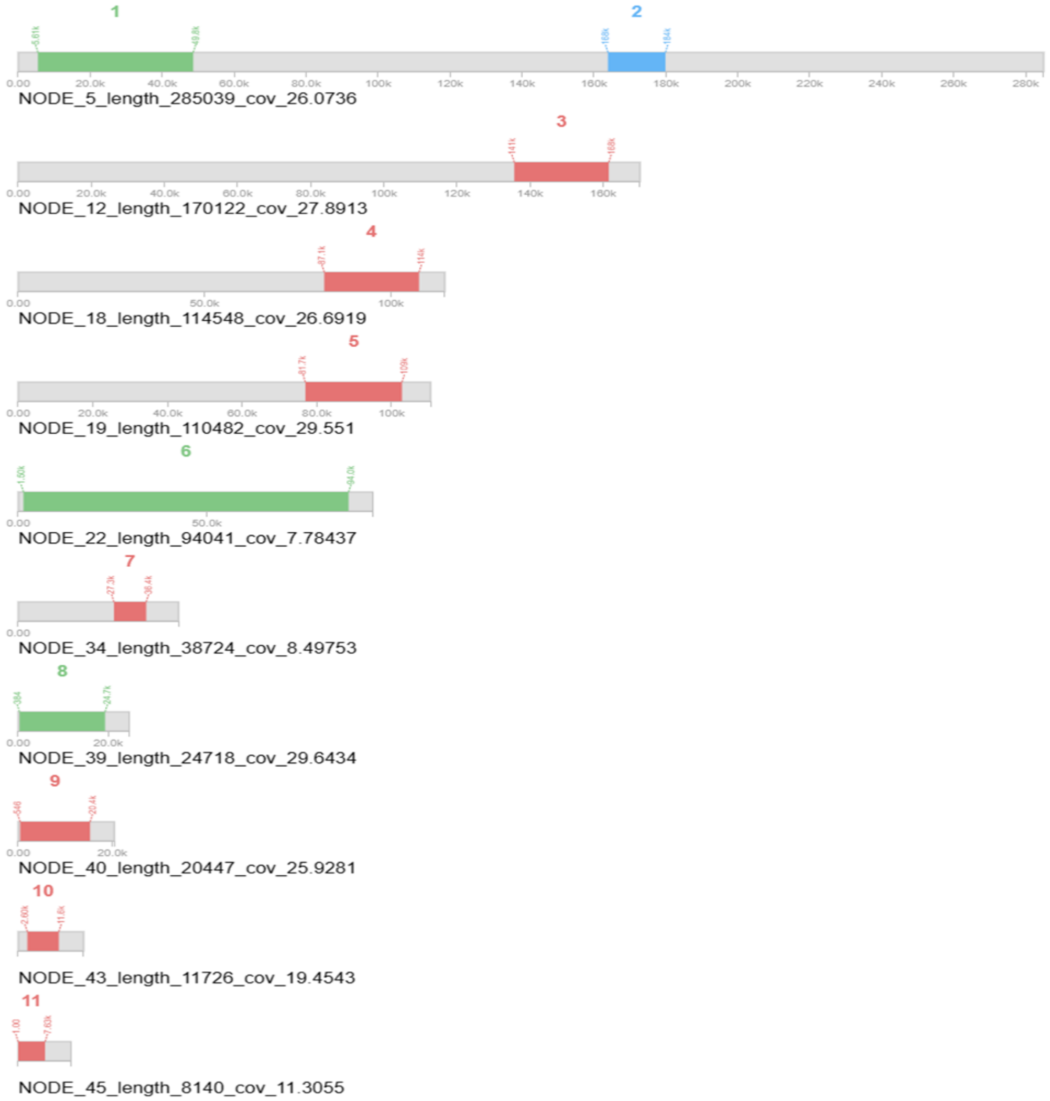

Appendix 4: Plasmid types containing resistance genes and their position in contigs

**Contigs**	**Resistance genes**	**Plasmid types**
**NODE_1_length_399443_cov_30.2848**	*ampC*1	IS5075
**NODE_2_length_314467_cov_36.7095**	*bac*A, *tol*C	MITEEc1
**NODE_3_length_298118_cov_33.439**	acrR	ISEc1
**NODE_7_length_249411_cov_31.6868**	*mdfA*	IS100
**NODE_7_length_249411_cov_31.6868**	*mdfA*	MITEEc1
**NODE_8_length_222074_cov_39.2814**	*ant*(3")-Ia, (*aad*A1), *dfr*A1	Tn7
**NODE_9_length_221455_cov_34.9789**	*amp*H,*mdt*, *sox*	ISEc1
**NODE_12_length_170122_cov_27.8913**	*emr*E (ethidium multidrug resistance protein E) (SMR protein family)	ISEc46PHAGE_Entero_SfI_NC_027339(6) partial
**NODE_13_length_161358_cov_37.5202**	*tet*(A), *ter*C	Tn5403
**NODE_25_length_73368_cov_37.4619**	-	MITEEc1
**NODE_38_length_25756_cov_32.6998**	-	ISSfl10
**NODE_43_length_11726_cov_19.4543**	*qnrS*1,*mph*(A), *bla*$_{CTX-M-15}$	ISKpn19
**NODE_45_length_8140_cov_11.3055**	-	IS629
**NODE_61_length_3370_cov_15.2439**	-	ISKox3
**NODE_65_length_3010_cov_7.26882**	*bla*$_{CTX-M-14}$	IS102
**NODE_66_length_2854_cov_39.711**	*aac*(3)-IIa	ISKpn19
**NODE_81_length_1657_cov_84.4856**	-	ISEc9
**NODE_93_length_1258_cov_75.9514**	-	IS3

Appendix 5: Mobile element finder results show the presence of alignment to the reference

Name	Synonyms	Type*	Allele length	e value	Identity	Coverage	Gaps	Substitution	Contig	Position in contig	Accession
**IS5075**	S110-like element	IS	1327	0	0.996232	1	0	5	NODE_1_length_399443_cov_30.2848	199338.. 200664	AM461_RS20090
**MITEEc1**		MIR	122	3.88E-54	0.99187	0.99187	1	0	NODE_2_length_314467_cov_36.7095	268267.. 268388	U00096
**ISEc1**	ISEc7, ISEc2, ISEc6, ISEc4, ISEc3	IS	1291	0	0.980635	1	0	25	NODE_3_length_298118_cov_33.439	162137.. 163427	HUZ68_RS06220,HUZ68_06160
**IS100**		IS	1954	0	0.995394	0.999488	1	8	NODE_7_length_249411_cov_31.6868	12833.. 14786	CP0045, Z32853
**MITEEc1**		MIR	123	8.56E-55	0.99187	1	0	1	NODE_7_length_249411_cov_31.6868	174628 174750	U00096
**Tn7**	TnC, Tn71, Tn72	UT	14067	0	1	1	0	0	NODE_8_length_222074_cov_39.2814	29980.. 44046	WP_011199102
**ISEc1**	ISEc7, ISEc2, ISEc6, ISEc4, ISEc3	IS	1291	0	0.963594	1	0	47	NODE_9_length_221455_cov_34.9789	180264.. 181554	HUZ68_RS06220,HUZ68_06160
**ISEc46**	IS200/IS605-like element	IS	1763	0	0.993761	1	0	11	NODE_12_length_170122_cov_27.8913	24546.. 26308	HUZ68_15635
**Tn5403**	Tn3-like element	UT	3664	0	0.999181	0.999727	1	2	NODE_13_length_161358_cov_37.5202	6105.. 9768	LGM30_RS27875, LGM30_27910
**MITEEc1**		MIR	123	2.55E-45	0.943089	1	0	7	NODE_25_length_73368_cov_37.4619	22084.. 22206	U00096
**ISSfl10**	IS3-like element	IS	1246	0	0.981437	0.987065	18	5	NODE_38_length_25756_cov_32.6998	3825.. 5070	WP_094105052
**ISKpn19**	ISKra4-like	IS	2851	0	1	1	0	0	NODE_43_length_11726_cov_19.4543	1780.. 4630	FY206_RS24740, FY206_24735
**IS629**		IS	1310	0	0.953435	1	0	61	NODE_45_length_8140_cov_11.3055	6704.. 8013	BAA31817.1, O82918
**ISKox3**	ISL3-like element	IS	1316	0	1	1	0	0	NODE_61_length_3370_cov_15.2439	1662.. 2977	AL524_RS00855, AL524_00055
**IS102**	isrC	IS	1057	0	0.926206	1	0	78	NODE_65_length_3010_cov_7.26882	907.. 1963	J01728
**ISEc9**	ISEcp1, ISEcp1B IS1380-like	IS	1656	0	1	1	0	0	NODE_81_length_1657_cov_84.4856	1.. 1656	DPF89_RS24060, DPF89_04760

	element										
**IS3**		IS	1258	0	0.99682	1	0	4	NODE_93_length_1258_cov_75.9514	1..1258	KPHS_05560

*Type: IS: Insertion sequence, MIR: Miniature inverted repeat, UT: unit transposon

Appendix 6: Phages found on contigs based on VirSorter v.2 2.2.4 tool using Proksee software

Contig	Group	shape	Max score
**NODE_5||0_partial**	dsDNAphage	linear	0.893
**NODE_5||1_partial**	dsDNAphage	linear	0.9
**NODE_12||0_partial**	dsDNAphage	linear	0.973
**NODE_18||0_partial**	dsDNAphage	linear	0.953
**NODE_22||full**	dsDNAphage	circular	0.967
**NODE_39||full**	dsDNAphage	linear	1
**NODE_40||full**	dsDNAphage	linear	0.993
**NODE_46||full**	dsDNAphage	linear	1
**NODE_47||full**	dsDNAphage	linear	0.993
**NODE_48||full**	dsDNAphage	linear	1
**NODE_49||full**	dsDNAphage	linear	1
**NODE_50||full**	dsDNAphage	linear	0.68
**NODE_51||full**	dsDNAphage	linear	0.933
**NODE_54||full**	dsDNAphage	linear	0.507
**NODE_55||full**	dsDNAphage	linear	0.953
**NODE_58||full**	dsDNAphage	linear	1
**NODE_59||full**	dsDNAphage	linear	0.78
**NODE_62||full**	dsDNAphage	linear	0.993
**NODE_63||full**	dsDNAphage	linear	0.887
**NODE_74||full**	ssDNA	linear	1
**NODE_80||full**	ssDNA	linear	0.973
**NODE_85||full**	ssDNA	linear	0.987
**NODE_86||full**	ssDNA	linear	0.993
**NODE_87||full**	ssDNA	linear	1
**NODE_91||full**	ssDNA	linear	1
**NODE_97||full**	ssDNA	linear	1
**NODE_101||full**	dsDNAphage	linear	1
**NODE_103||full**	dsDNAphage	linear	0.98
**NODE_131||full**	ssDNA	linear	0.913
